# Regulation of Mitochondrial Energy Metabolism by Glutaredoxin 5 in the Apicomplexan Parasite Neospora caninum

**DOI:** 10.1128/spectrum.03091-22

**Published:** 2022-12-21

**Authors:** Xingju Song, Xu Yang, Zhu Ying, Kaijian Wu, Jing Liu, Qun Liu

**Affiliations:** a National Animal Protozoa Laboratory and Key Laboratory of Animal Epidemiology of the Ministry of Agriculture, College of Veterinary Medicine, China Agricultural University, Beijing, China; b College of Animal Science and Technology, Guangxi University, Nanning, China; Hubei University of Medicine

**Keywords:** *Neospora caninum*, glutaredoxin 5, iron-sulfur cluster assembly, energy metabolism

## Abstract

Iron-sulfur [Fe-S] clusters are one of the most ancient and functionally versatile natural biosynthetic prosthetic groups required by various proteins involved in important metabolic processes, including the oxidative phosphorylation of proteins, electron transfer, energy metabolism, DNA/RNA metabolism, and protein translation. Apicomplexan parasites harbor two possible [Fe-S] cluster assembly pathways: the iron-sulfur cluster (ISC) pathway in the mitochondria and the sulfur formation (SUF) pathway in the apicoplast. Glutaredoxin 5 (GRX5) is involved in the ISC pathway in many eukaryotes. However, the cellular roles of GRX5 in apicomplexan parasites remain to be explored. Here, we showed that Neospora caninum mitochondrial GRX5 (NcGRX5) deficiency resulted in aberrant mitochondrial ultrastructure and led to a significant reduction in parasite proliferation and virulence in mice, suggesting that NcGRX5 is important for parasite growth *in vitro* and *in vivo*. Comparative proteomics and energy metabolomics were used to investigate the effects of NcGRX5 on parasite growth and mitochondrial metabolism. The data showed that disruption of NcGRX5 downregulated the expression of mitochondrial electron transport chain (ETC) and tricarboxylic acid cycle (TCA) cycle proteins and reduced the corresponding metabolic fluxes. Subsequently, we identified 23 proteins that might be adjacent to or interact with NcGRX5 by proximity-based protein labeling techniques and proteomics. The interactions between NcGRX5 and two iron-sulfur cluster synthesis proteins (ISCS and ISCU1) were further confirmed by coimmunoprecipitation assays. In conclusion, NcGRX5 is important for parasite growth and may regulate mitochondrial energy metabolism by mediating the biosynthesis of iron-sulfur clusters.

**IMPORTANCE** Iron-sulfur [Fe-S] clusters are among the oldest and most ubiquitous prosthetic groups, and they are required for a variety of proteins involved in important metabolic processes. The intracellular parasites in the phylum *Apicomplexa*, including Plasmodium, Toxoplasma gondii, and Neospora caninum, harbor the ISC pathway involved in the biosynthesis of [Fe-S] clusters in mitochondria. These cofactors are required for a variety of important biological processes. However, little is known about the role of oxidoreductase glutaredoxins in these parasites. Our data indicate that NcGRX5 is an essential protein that plays multiple roles in several biological processes of N. caninum. NcGRX5 interacts with the mitochondrial iron-sulfur cluster synthesis proteins ISCS and ISCU1 and also regulates parasite energy metabolism. These data provide an insider’s view of the metabolic regulation and iron-sulfur cluster assembly processes in the apicomplexan parasites.

## INTRODUCTION

Neospora caninum is an obligate intracellular apicomplexan parasite that causes neosporosis, which has become an international concern due to the association between parasitic infections and abortions in dairy and beef cattle (1–3). N. caninum shares similar morphology, host range, and clinical symptoms with Toxoplasma gondii (1). Investigations to determine whether the pathogen poses a potential threat to human health are ongoing. Vaccines and drugs are urgently needed for prevention and treatment, as serological identification in several countries has shown positive antibodies against N. caninum in humans; however, viable parasites have not yet been isolated. Glutaredoxin (GRX) is a small thiol-disulfide oxidoreductase that receives electrons directly from the donor glutathione (GSH) and is important for the intracellular thiol redox system (4). The types of GRX proteins and their distribution patterns vary from species to species. In humans, there are four types of GRXs, which are located in cytoplasm, nucleus, and mitochondria. In yeast, there are seven types of GRXs, located in the cytoplasm, nucleus, mitochondria, and endoplasmic reticulum/Golgi apparatus (4). Studies on parasites have mainly focused on cytoplasmic GRX1, and mitochondrial GRX5 has been less studied. Trypanosoma brucei glutaredoxin 2 (GRX2) is a dithiol glutaredoxin located specifically in the mitochondrial intermembrane space. In the procyclic stage, TbGRX2 deficiency significantly affects the morphology of parasites and leads to irreversible proliferation arrest (5). Growth arrest is reduced at high glucose concentrations, which suggests that GRX2 may be involved in the regulation of a respiratory chain component (5).

[Fe-S] clusters are protein cofactors that are required for various biological processes, including the oxidative phosphorylation of proteins, electron transfer, energy metabolism, DNA/RNA metabolism, and protein translation (6–8). The crystal structure of human GLRX5 was analyzed, and the results showed that GLRX5 binds to two [2Fe-2S] clusters and four GSH molecules, implying that GLRX5 is an iron-sulfur cluster synthesis and transport protein (9). Yeast GRX5 physically interacts with the Hsp70 chaperone SSQ1 at a site different from its interaction with ISCU1 and accepts [2Fe-2S] clusters from ISCU1 (8, 10). The absence of a chaperone system in the membrane or GRX5 leads to the accumulation of [Fe-S] clusters on ISCU1, which may affect the downstream target [Fe-S] proteins that typically receive [2Fe-2S] clusters (8). The [Fe-S] cluster binding ability of GRXs has been identified in trypanosomes and Leishmania protozoa (11, 12). However, the roles of GRX5 in [Fe-S] protein synthesis in apicomplexan parasites remain unclear.

Our previous study identified five GRXs in N. caninum localized in different compartments. Two cytoplasmic NcGRXs (NcGRX1 and NcGRX3) and two apicoplast NcGRXs (NcGRX S14 and NcGRX C5) have been identified in our previous studies (13, 14). In this study, we identified the GRX5 protein in N. caninum (NcGRX5) and explored the role of NcGRX5 in [Fe-S] protein assembly and energy metabolism. Our results indicate that NcGRX5 is located in the mitochondria of N. caninum tachyzoites and is important for parasite growth *in vitro* and *in vivo*. NcGRX5 deficiency affects electron transport chain (ETC) and tricarboxylic acid cycle (TCA) pathway proteins and alters the corresponding metabolic products. In addition, we demonstrated that NcGRX5 interacts with [Fe-S] cluster synthesis proteins (ISCS and ISCU1).

## RESULTS

### Sequence characterization and phylogenetic analysis of NcGRX5.

**FIG 1:**
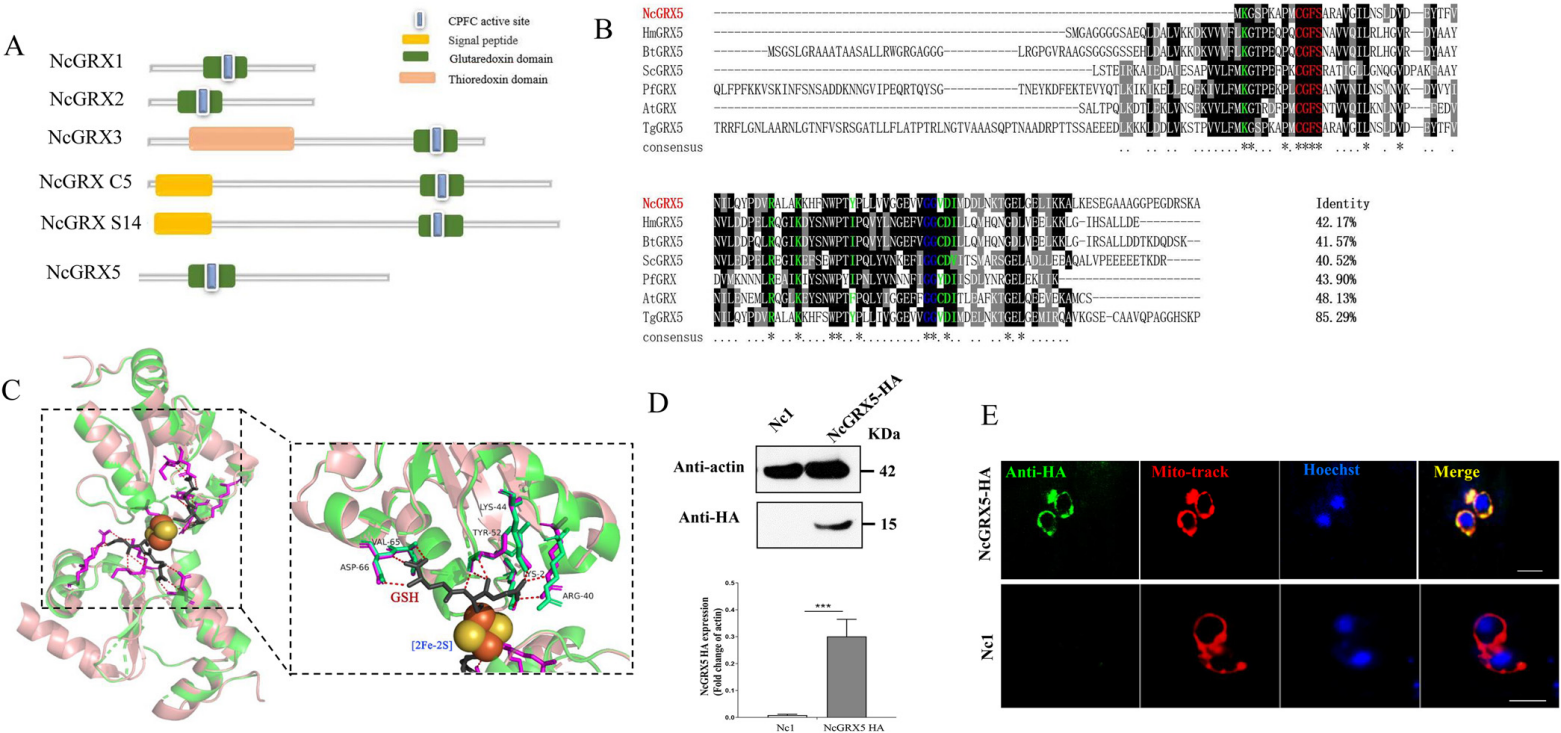
Characterization of the structures, activities, and locations of NcGRX5. (A) The model pattern of all glutaredoxin proteins in Neospora caninum. The active sites consist of amino acid residues Cys-Pro-Phe-Cys (CPFC) were conserved in the six GRXs. (B) Process of protein sequence alignment of NcGRX5 with homologues in other species using Clustal X. The percentage of NcGRX5, which is homologous to other GRXs, is shown at the end of the alignment. The active-site residues CGFS are marked with a red letter. Residues interacting with GSH are marked with a green letter. The conserved Gly-Gly site is marked with a blue letter. (C) Performance of a three-dimensional structural modeling based on the crystalline structure of H. sapiens GRX5 (PDB code 2WUL). PyMOL was used to mark possible GSH-binding sites and select pocket coordinate positions on the three-dimensional structures. The key amino acid residues in NcGRX5 interacting with GSH were predicted by AutoDock Vina and are highlighted in purple (Lys^2^, Arg^40^, Lys^44^, Tyr^52^, Val^65^, and Asp^66^). (D) Western blot analysis confirmed expression of hemagglutinin (HA)-tagged NcGRX5 in parasites using mouse HA antibody. Actin was used as a control. The expression of HA-tagged NcGRX5 protein was quantitatively evaluated by ImageJ based on three independent experiments. Statistical analysis was performed using one-way analysis of variance (ANOVA). ***, *P < *0.001. ND, not detected. (E) Intracellular parasite NcGRX5-HA. The cellular localization of NcGRX5 was analyzed by immunofluorescence assays using NcGRX5-HA parasites. Parasites were fixed and stained for anti-HA (green) and MitoTracker (red). MitoTracker is considered a mitochondrial marker. Nc1 parasites were used as a negative control to confirm the specificity of the HA signal. Bar, 5 μm.

We identified five glutaredoxin-containing genes of N. caninum in the ToxoDB genomic resource database (Fig. 1A). NcGRX1 and NcGRX3 are located in the cytoplasm (13). Similarly, NcGRX S14 and NcGRX C5 are located in the apicoplast (14). Since N. caninum belongs to eukaryotes, the number and location of GRXs differ greatly between prokaryotes and eukaryotes. Thus, sequence homology alignment of NCLIV_037620 with eukaryotes showed a similarity to glutaredoxin 5 in Homo sapiens (42.17%) and Saccharomyces cerevisiae (40.52%), which was finally named NcGRX5. Sequence analysis showed that NcGRX5 has a CGFS active site, a classical monothiol motif, in the glutaredoxin domain (Fig. 1B). Sequence alignment revealed that NcGRX5 contains GSH-binding motifs (residues Lys^2^, Arg^40^, Lys^44^, Tyr^52^, Val^65^, and Asp^66^) (Fig. 1B). The crystal structure shows that human GRX5 binds to two [2Fe-2S] clusters and four GSH molecules to form a tetramer. Each [2Fe-2S] cluster is ligated by N-terminal cysteine (Cys^67^) thiols contributed by two GRX5 proteins and two cysteine thiols contributed by two GSH proteins (Fig. 1C) (9). Since NcGRX5 exhibits sequence homology (42.17%) to that of human GRX5, we use the structural model of H. sapiens GRX5 (HsGRX5, PDB code 2WUL) as a template to predict the three-dimensional (3D) structure of NcGRX5 and found that the 3D structure almost completely overlaps (Fig. 1C).

To determine the localization of NcGRX5, we constructed hemagglutinin (HA) epitope-tagged NcGRX5 in a wild-type (WT) parasite strain (Nc1) of N. caninum (Fig. S1A). Western blot analysis with an anti-HA antibody showed a single band of the expected size of the NcGRX5 protein (Fig. 1D). Immunofluorescence assays (IFAs) show that NcGRX5 is located in the mitochondrion (Fig. 1E).

### Importance of NcGRX5 for the growth of N. caninum.

**FIG 2:**
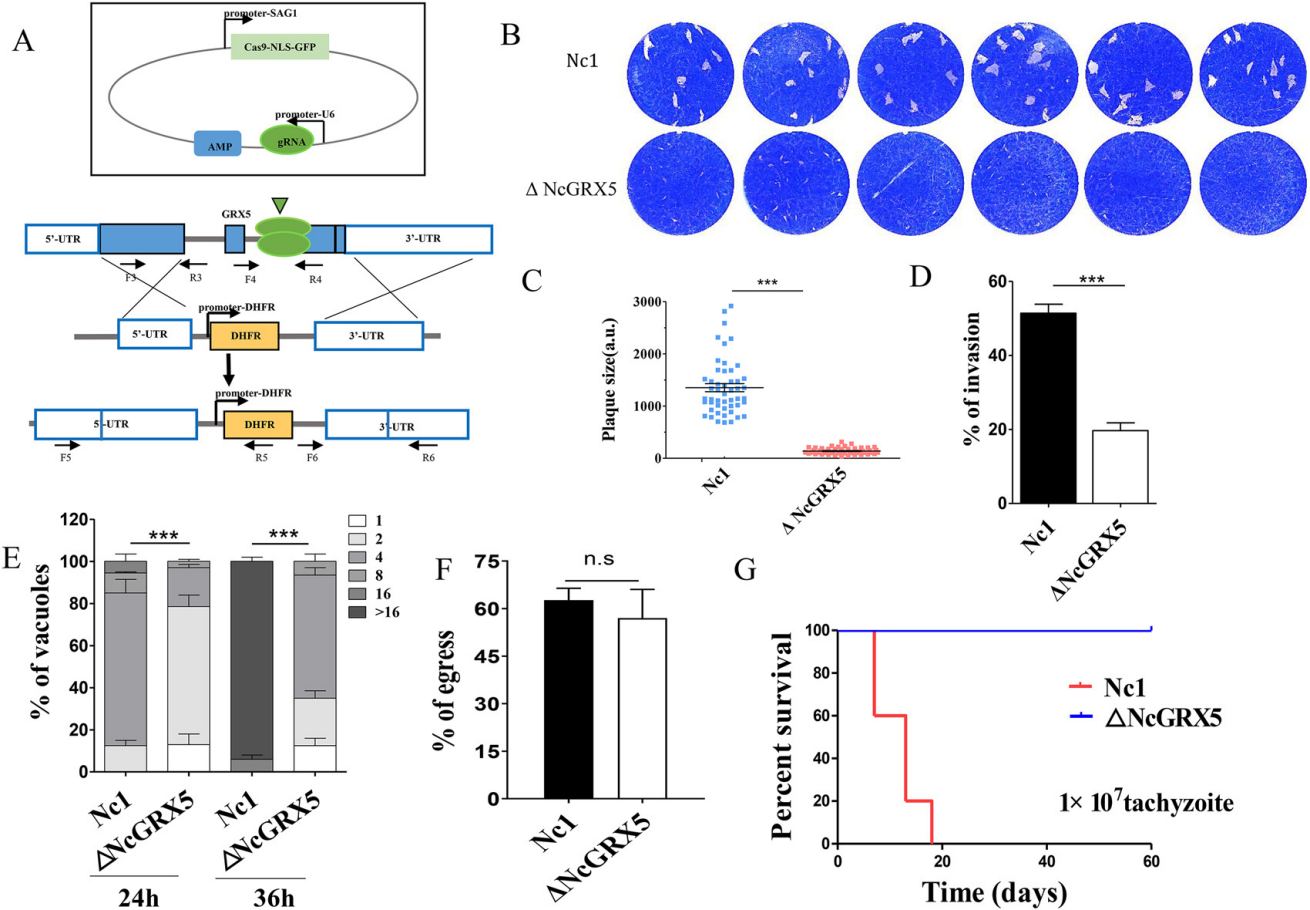
Reduction in the growth of parasites due to lack of NcGRX5. (A) Strategy for constructing the ΔNcGRX5 strain. (B) Comparing the overall growth ability of wild-type and knockout parasites using plaque assays. Each well was infected with 300 parasites, and plaques were stained for 9 days. (C) Measurements of plaque areas using pixel points in Photoshop C6S software (Adobe, USA). The data were compiled from three independent experiments. Plaque areas were analyzed using one-way ANOVA with Tukey’s *post hoc* test. Asterisks indicate a significant difference. ***, *P* < 0.001. (D) Inoculation of HFFs with 1 × 10^5^ parasites and continuous culture for 24 h. The invasion ratio is expressed as the number of vacuoles per host cell. SRS2 was used as a parasite membrane marker, and Hoechst dye was used to count the number of cells. The data are expressed as means ± standard error of the mean (SEM) based on three assays and *t* test analysis. (E) Inoculation of Nc1 and ΔNcGRX5 tachyzoites (1 × 10^5^) into HFFs and growth for 24 h to evaluate their intracellular replication ability. The data are expressed as the means ± SEM of results obtained from three assays, and in each assay, 100 total PVs of each strain were counted. The data were analyzed by two-way ANOVA. (F) Assessment of parasite egress capacity after treatment with calcium ionophore A23187. The ratio of 100 randomly selected ruptured/integrated vacuoles was determined for each slide. (G) Survival rate of mice after infection with different strains. A total of 1 × 10^7^ tachyzoites were injected intraperitoneally into BALB/c mice (*n* = 5). Statistical analysis was performed using the LIFETEST procedure in the Statistical Analysis System software (SAS Institute Inc., USA). gRNA, genomic RNA; n.s, no significant difference; UTR, untranslated region.

To investigate the function of NcGRX5, we generated a knockout strain (ΔNcGRX5) using the CRISPR/Cas9 system (Fig. 2A). The establishment of fully NcGRX5-deficient parasites was validated by PCR (Fig. S1B). To assess the viability of the NcGRX5-deficient parasite, we monitored the formation of plaques during a continuously maintained 9-day culture. A significant reduction (*P < *0.001) in plaque formation area was observed in ΔNcGRX5 parasites compared to Nc1 parasites (Fig. 2B and C).

To further explore the reasons for the reduction in plaque area, we investigated the biological role of NcGRX5 in the lytic cycle of N. caninum. As previously reported, the complete lytic cycle of tachyzoite growth in N. caninum includes invasion, intracellular replication, and egress stages. Disruption of one or more of these processes reduces the plaque area (15, 16). The results showed that host cell invasion by ΔNcGRX5 parasites was significantly impaired (~60%, *P < *0.001) compared to Nc1 parasites (Fig. 2D). Intracellular replication of the NcGRX5-deficient parasites was also notably reduced (Fig. 2E). No significant difference was observed in the egress ability of ΔNcGRX5 and Nc1 parasites (Fig. 2F). To assess the contribution of NcGRX5 to parasite growth *in vivo*, BALB/c mice (five mice of each strain) were injected with ΔNcGRX5 or Nc1 parasites. All mice infected with 1 × 10^7^ Nc1 parasites died within 20 days. All mice infected with the same dose of ΔNcGRX5 parasites survived for 60 days (Fig. 2G). These results suggest that NcGRX5 is critical for the growth of N. caninum *in vivo* and *in vitro*.

### Complementation of NcGRX5 restored the growth phenotype of parasites.

**FIG 3:**
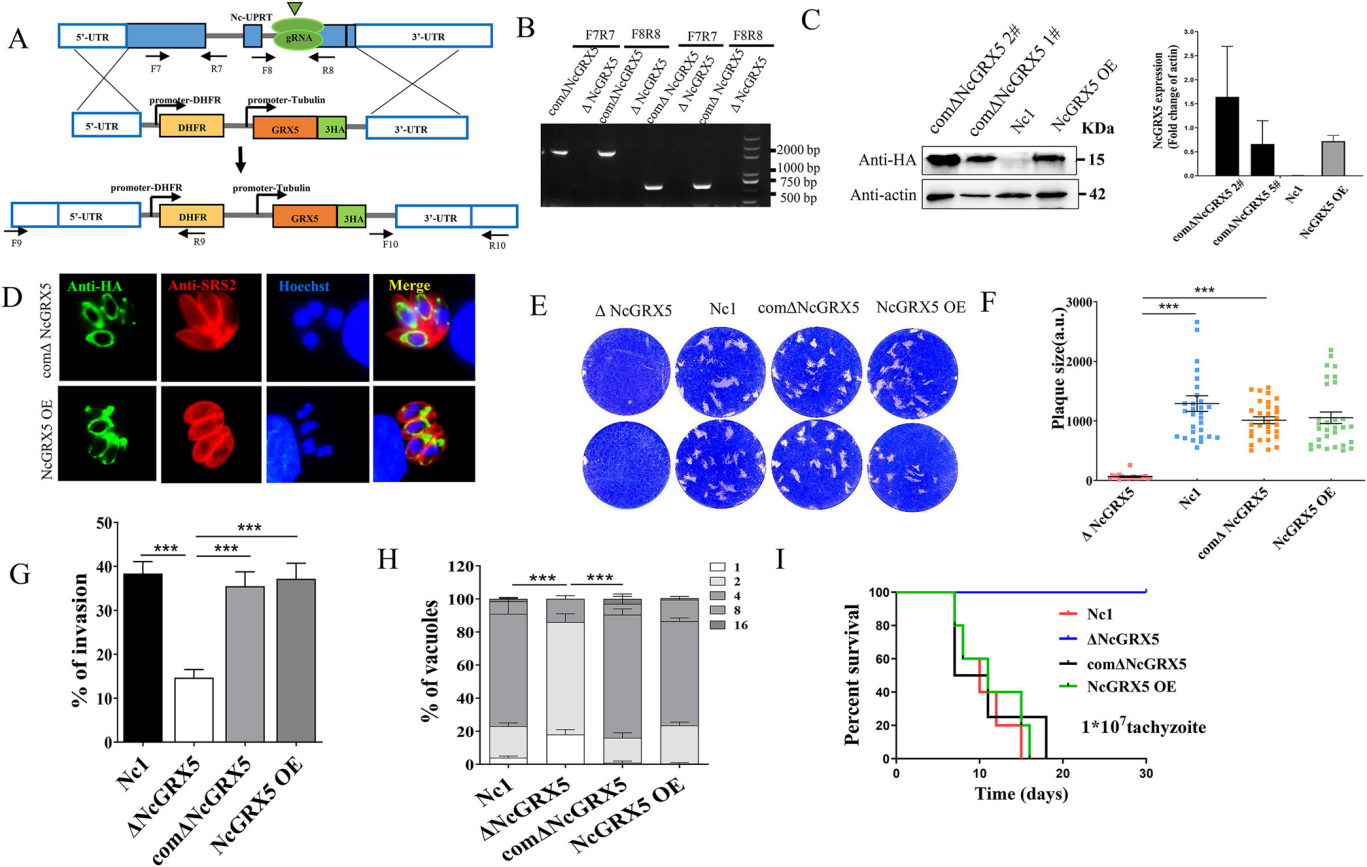
Possible restoration of the growth phenotype by complementing the NcGRX5 gene in ΔNcGRX5 parasites. (A) Construction strategy for complementation parasites (comΔNcGRX5) and overexpression parasites (NcGRX5 OE). (B) Confirmation of NcGRX5 OE and comΔNcGRX5 parasites by PCR. (C) Confirmation of NcGRX5 OE and comΔNcGRX5 parasites by Western blotting using mouse anti-HA antibody. Actin was used as a loading control. The immunoblotting band was quantitatively evaluated by ImageJ based in two independent experiments. The error bars represent the standard error. (D) Confirmation of NcGRX5 OE and comΔNcGRX5 parasites by IFA. The location of NcGRX5 (green) was visualized with anti-HA, and SRS2 (red) was used as a parasite shape marker. (E) Comparing the overall growth ability of WT, ΔNcGRX5, NcGRX5 OE, and iΔNcGRX5 parasites using plaque assays. (F) Measurements of plaque areas using the pixel point in Photoshop C6S software (Adobe, USA). The data are derived from three independent experiments. (G) Comparison of the invasion ability of comΔNcGRX5, ΔNcGRX5, NcGRX5 OE, and wild-type (WT) parasites. (H) Comparison of the intracellular replication ability of comΔNcGRX5, ΔNcGRX5, NcGRX5 OE, and WT parasites. (I) Survival rate of mice after infection with 1 × 10^7^ comΔNcGRX5, ΔNcGRX5, NcGRX5 OE, and WT parasites. DHFR, dihydrofolate reductase.

Complementation (iΔNcGRX5) and overexpression (NcGRX5 OE) of NcGRX5 was achieved by replacing the UPRT gene locus in ΔNcGRX5 and Nc1 parasites with the NcGRX5-HA expression cassette (Fig. 3A), and successful cassette replacement was validated by PCR analysis, Western blotting, and IFA (Fig. 3B to D). Phenotype assays revealed that the plaque-forming area was restored in iΔNcGRX5 parasites (Fig. 3E and F) and that overexpression did not affect the plaque-forming area. Similarly, complementation of NcGRX5 restored the invasion and intracellular replication ability of these parasites (Fig. 3G and H). The growth of iΔNcGRX5 and NcGRX5 OE parasites was not significantly different from that of Nc1 parasites but was significantly different from that of ΔNcGRX5 parasites (Fig. 3I). These results suggest that complementation of NcGRX5 restores the growth phenotype of the parasites and that overexpression of NcGRX5 does not affect the growth phenotype of the parasites.

### Loss of NcGRX5 results in aberrant mitochondrial morphology.

**FIG 4:**
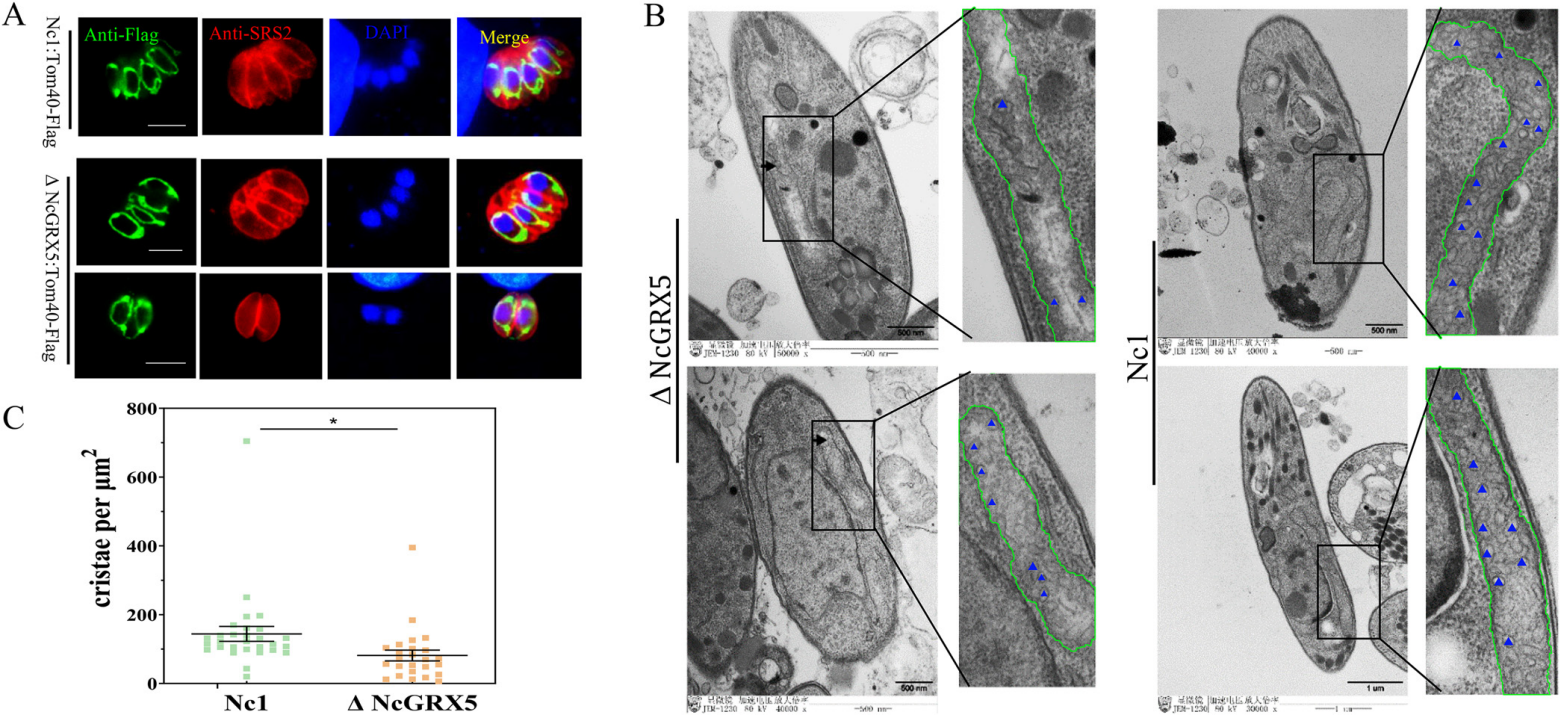
Loss of NcGRX5 results in aberrant mitochondrial morphology. (A) Addition of FLAG tags to the C terminus of the endogenous outer mitochondrial membrane protein TOM40 in ΔNcGRX5 and Nc1 parasites. The outer mitochondrial membrane morphology was visualized by IFA. SRS2 (red) is considered a parasite shape marker. TOM40 (green) is considered a parasite outer mitochondrial membrane marker. Bar, 5 μm. (B, C) Electron micrographs of ΔNcGRX5 and Nc1 parasites. Insets show representative mitochondria. Mitochondria are outlined by green lines. Parts of the cristae are marked with blue triangles. The number of cristae per μm^2^ was statistically analyzed. The values are the means ± standard deviation (SD). *, *P = *0.027. DAPI, 4′,6-diamidino-2-phenylindole.

Considering the importance of NcGRX5 for parasite growth and its mitochondrial localization, we sought to determine whether this protein affects mitochondrial morphology and function. Using CRISPR-mediated endogenous tagging, we introduced a C-terminal FLAG epitope into TOM40, an outer mitochondrial membrane marker in ΔNcGRX5 and Nc1 parasites. Immunofluorescence results showed a lasso-like pattern of distribution of TOM40 around the nucleus of ΔNcGRX5 and Nc1 parasites (Fig. 4A), which is typical of the distribution of TOM40 in the mitochondria of apicomplexan parasites (17). To further observe the ultrastructure of mitochondria in ΔNcGRX5 and Nc1 parasites, transmission electron microscopy was performed. The mitochondria of Nc1 parasites presented a regular morphology including an intact cristae structure (Fig. 4B). The deletion of NcGRX5 resulted in a loss of mitochondrial density and a reduction in the number of mitochondrial cristae (Fig. 4B and C). The outer mitochondrial membrane in ΔNcGRX5 parasites was not affected (Fig. 4B). These results suggest that NcGRX5 deficiency triggers the acquisition of aberrant morphology and a reduced number of mitochondrial cristae in N. caninum.

### Significant downregulation of ETC and TCA cycle proteins due to NcGRX5 deficiency.

**FIG 5:**
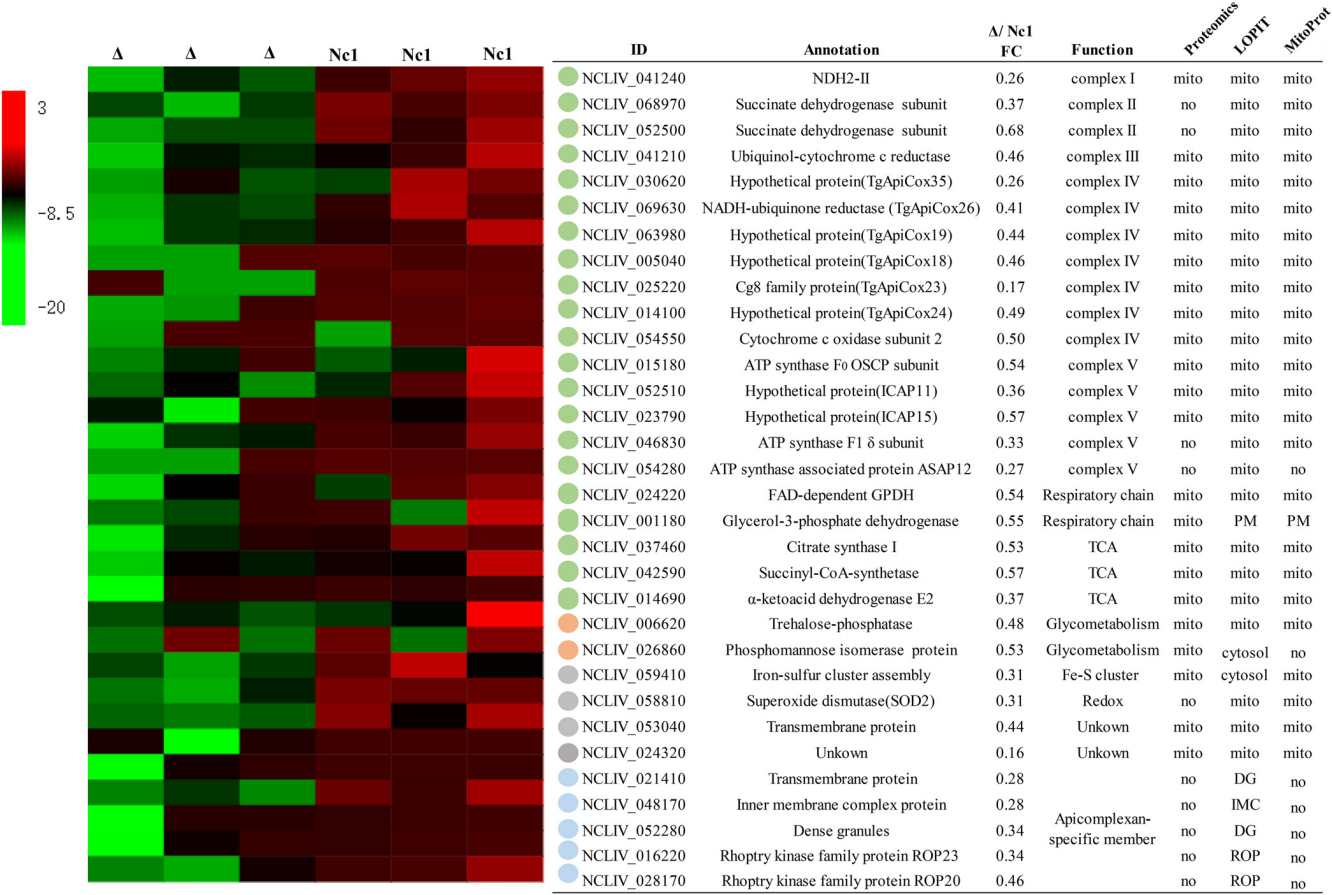
Comparative proteomic analysis of ΔNcGRX5 and Nc1 parasites. Comparative proteomic analysis was performed to determine the expression levels of all proteins in Nc1 and ΔNcGRX5 parasites. Equal numbers of ΔNcGRX5 and Nc1 parasites were used for proteomic analysis. The samples were analyzed by liquid chromatography-tandem mass spectrometry (LC-MS/MS) for “label-free” quantification using PEAKS software. Proteins with absolute fold changes ≥1.5 (*P < *0.05, *t* test) were considered significantly downregulated proteins (see details in Data set 1). The locations of these downregulated proteins were analyzed using T. gondii mitochondrial proteomic data, as well as MitoProt and LOPIT. Possible mitochondrial proteins and Apicomplexan-specific proteins were selected and annotated via ToxoDB search. A heat map of expression values is shown for selected proteins in Nc1 and ΔNcGRX5 parasites. The proteins were clustered according to function. The mean log_2_ gene expression values are presented. CoA, coenzyme A; TCA, tricarboxylic acid cycle; PM, plasma membrane; DG, dense granule; IMC, inner membrane complex; ROP, rhoptry; ICAP, indispensable conserved apicomplexan proteins.

The morphology of mitochondria is closely related to their functions. The MICOS complex on the mitochondrial inner boundary membrane (IBM) belongs to a family of proteins that orchestrate mitochondrial morphology and dynamics (18). The mitochondrial electron transport chain, especially ATP synthase (complex V), has also been shown to be associated with cristae shape (18, 19). Consistently, it has been clearly shown that cristae shape affects ETC performance and complex formation (18). Therefore, it is interesting to investigate whether deletion of GRX5 affects the mitochondrial pathways in N. caninum. A comparative proteomic analysis showed that a total of 247 proteins were downregulated in ΔNcGRX5 parasites compared to Nc1 parasites, and Gene Ontology (GO) enrichment analysis revealed that these proteins participate in mitochondrial inner membrane, ETC, and superoxide dismutase activity processes (Fig. S2A to D; Data set S1). These downregulated proteins were subsequently screened based on homology, location, and function (Fig. 5). The location of the protein was predicted using T. gondii mitochondrial proteomic data (20), MitoProt and hyperplexed localization of organelle proteins by isotope tagging (hyperLOPIT) data (21, 22). Among these proteins, 23 target proteins were predicted as mitochondrial proteins, and 5 were identified as apicomplexan-specific proteins. Cluster analysis based on the function of the target proteins showed that disruption of NcGRX5 mainly resulted in downregulation of proteins involved in the mitochondrial ETC and TCA cycle pathways (Fig. 5). Furthermore, several apicomplexan-specific member proteins were affected.

### Influence of NcGRX5 on mitochondrial energy metabolism fluxes.

**FIG 6:**
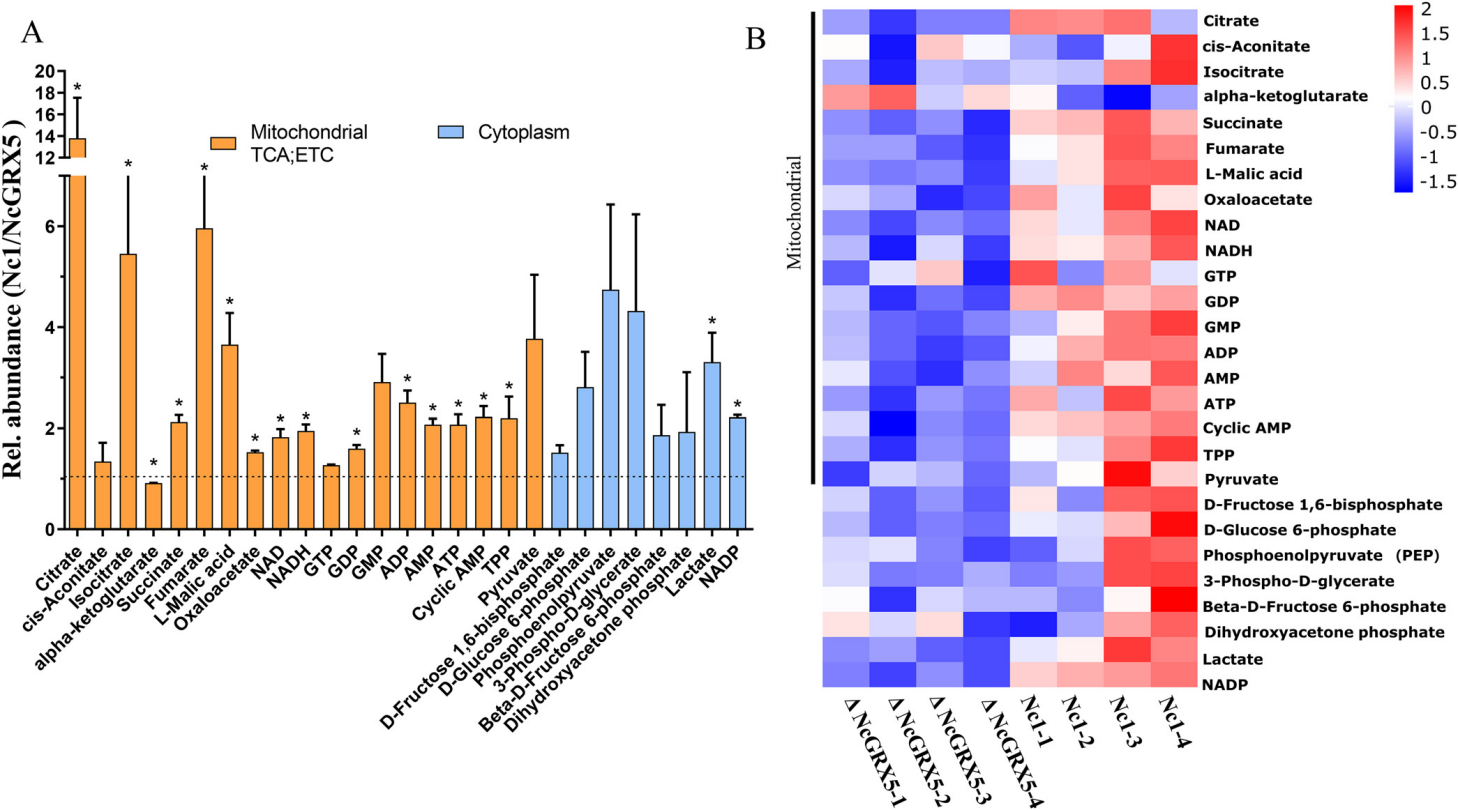
Influence of NcGRX5 on energy metabolism fluxes. (A) Relative (%) abundance of selected metabolites in Nc1 and ΔNcGRX5 parasites. Bars represent the abundance of metabolites in Nc1 parasites compared to ΔNcGRX5 parasites. Mitochondrial metabolites are indicated by orange columns, and cytoplasmic metabolites are indicated by blue columns. All metabolites were measured by MS-MS, as detailed in Data set 2. (B) Heat map of absolute abundance of metabolites in Nc1 and ΔNcGRX5 parasites. Mean log_2_ gene expression values are presented. Mitochondrial metabolites are marked on the left side of the heat map. The data are presented as means ± SEM of four independent experiments. *, *P < *0.05, *t* test. ETC, electron transport chain; TPP, thiamin pyrophosphate.

We further compared the energy metabolite levels of Nc1 and ΔNcGRX5 tachyzoites by performing liquid chromatography-mass spectrometry (LC-MS) (Data set S2). We found a significant reduction in TCA cycle metabolites (citrate, isocitrate, succinate, fumarate, and l-malic acid), ETC metabolites (succinate, NAPH/NAD, NADP, ADP, ATP, AMP, and cyclic AMP), glycolysis production (lactate), and thiamin pyrophosphate (TPP, a cofactor in the synthesis of acetyl-coenzyme A [acetyl-CoA] from pyruvate) in ΔNcGRX5 parasites compared to the corresponding levels in the Nc1 control strain (Fig. 6A and B). Citrate is produced by acetyl-CoA upon catalysis of citrate synthase, whereas succinate is produced by succinyl-CoA upon catalysis of succinyl-CoA-synthetase. Reduced expression and activity of citrate synthase and succinyl-CoA-synthetase resulted in a significant decrease in the corresponding metabolites (citrate and succinate) and could further affect the downstream metabolites (isocitrate, succinate, fumarate, and l-malic acid) (Fig. 5 and 6A). In addition, ATP was significantly reduced, which might be due to the decrease of certain mitochondrial ETC proteins (Fig. 5). The decrease in ATP is usually accompanied by an increase in ADP or AMP; however, the reason for the simultaneous decrease in ADP and AMP in ΔNcGRX5 tachyzoites should be further studied. Taken together, these results suggest that disruption of NcGRX5 affects the mitochondrial respiratory chain complex and TCA cycle pathways and alters the corresponding metabolic fluxes.

### Identification of candidate NcGRX5-interacting proteins using the BioID method.

**FIG 7:**
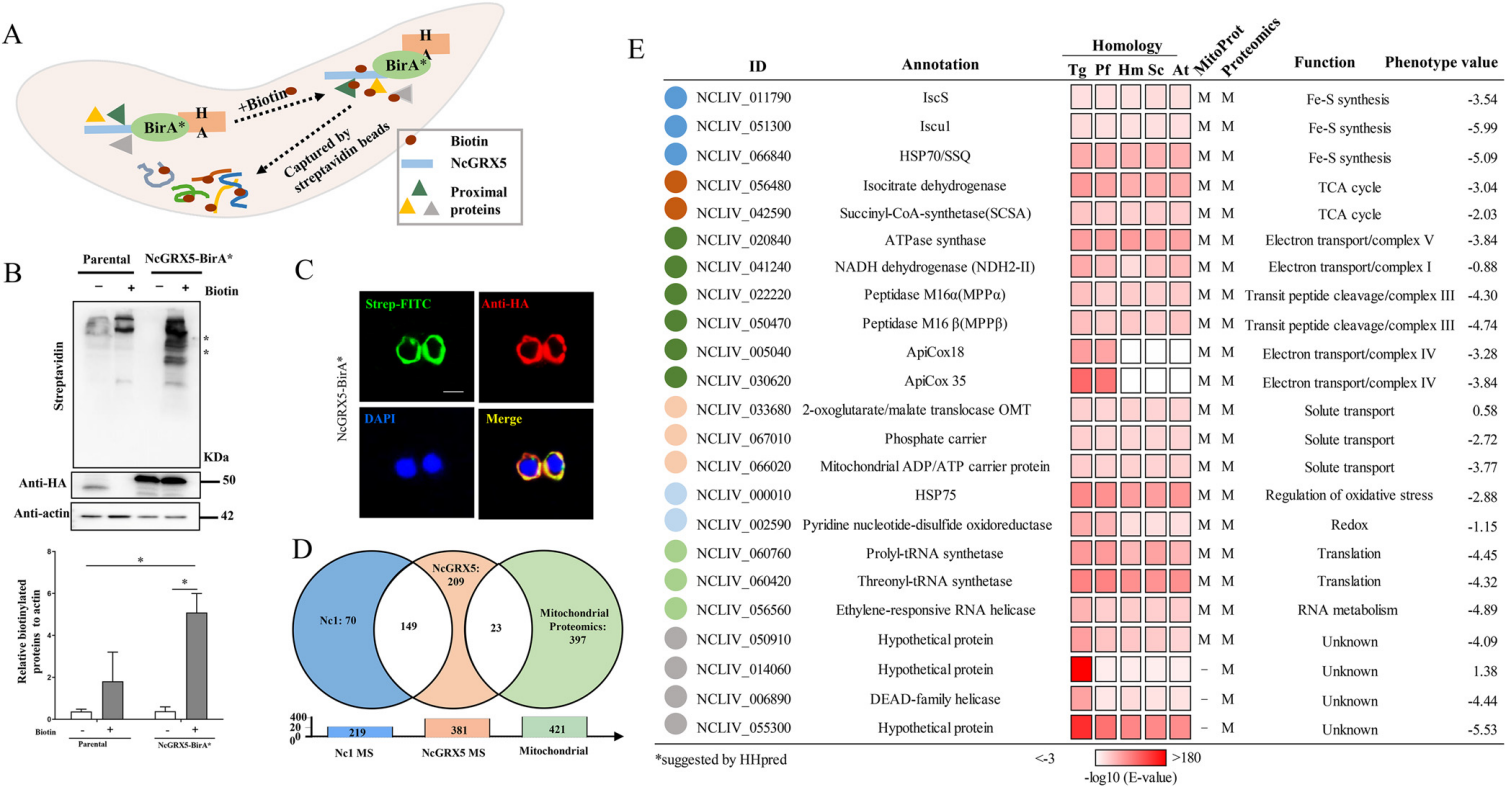
Candidate NcGRX5-interacting proteins identified by the BioID method. (A) Diagram showing the BioID method. NcGRX5-BirA* is expressed in N. caninum. Biotinylated proteins were captured with streptavidin beads. (B) Western blot detection of biotinylated proteins in parental and NcGRX5-BirA*-expressing parasites ± biotin treatment. Lysates were probed with streptavidin-HRP, revealing an increase in biotinylated proteins in NcGRX5-BirA* parasites following biotin addition. The NcGRX5-BirA* fusion protein is expected to be ~50 kDa (arrowhead). Actin was used as a loading control. The biotinylated protein level normalized to β-actin was quantified by ImageJ from two independent experiments. Error bars represent standard errors. A one-way ANOVA was performed with Tukey’s correction for multiple comparisons *, *P = *0.0452. (C) Immunofluorescence assay detection of biotinylated proteins in the NcGRX5-BirA* strain using streptavidin-FITC (green) and colocalization with NcGRX5. The location of NcGRX5 was detected by α-HA antibodies (red). Bar, 5 μm. (D) LC-MS was performed with 219 biotinylated proteins identified in parental parasites and 381 in NcGRX5-BirA*-expressing parasites. A total of 149 common nonspecific proteins were identified and removed. The remaining 232 proteins in NcGRX5-BirA*-expressing parasites correspond to the T. gondii mitochondrial proteome, with only 23 candidate proteins located in the mitochondria. (E) Table showing the 23 candidate biotinylated proteins located in the mitochondria (for more information, see Data set 3). The possible localization of proteins located in mitochondria (M) was evaluated using the T. gondii Mitochondrial Proteome Database and predicted using MitoProt (20–22). These proteins were annotated via ToxoDB searches or sequence homology alignment with T. gondii (Tg), P. falciparum (Pf), H. sapiens (Hs), S. cerevisiae (Sc), and A. thaliana (At). Homology alignment was performed by HHpred using a hidden Markov model-based search. The phenotype value was obtained from the corresponding T. gondii homologous protein in ToxoDB.

Considering the above-mentioned findings, to explore whether NcGRX5 directly interacts with proteins to affect downstream energy metabolism pathways, we utilized the BioID technique to screen NcGRX5-interacting proteins (20, 23). The biotin ligase BirA* and 3HA epitope tags were fused to the C terminus of NcGRX5 (Fig. 7A), and NcGRX5 was used as bait to capture biotinylated proteins, the success of which was verified by Western blotting (Fig. 7B). Colocalization of biotinylated proteins with NcGRX5 was determined by IFA and compared with results obtained with Nc1 parasites (Fig. 7C).

Biotinylated proteins from NcGRX5-BirA* and Nc1 parasites were purified using streptavidin magnetic beads and analyzed by LC-MS/MS. A total of 381 proteins were identified in the NcGRX5-BirA* parasite, of which 219 were proteins not specific to Nc1 parasites (Data set S3). A total of 232 unique proteins were observed in the NcGRX5-BirA* parasite, but not in the Nc1 parasite (Fig. 7D; Data set S3). Twenty-three possible mitochondrial proteins were screened by using the T. gondii mitochondrial proteome data (Fig. 7D) (20) and predicted using the MitoProt program (Fig. 7E) (21). Using the protein function and structure prediction server HHpred, we compared homologues of 23 candidate proteins in model organisms (T. gondii, Plasmodium falciparum, H. sapiens, S. cerevisiae, and Arabidopsis thaliana) (24). The results showed that the candidate proteins except ApiCox18 and ApiCox15 were homologous to the corresponding proteins in the model organism (*E* value < 10^−5^) (Fig. 7E). ApiCox18 and ApiCox15 had corresponding homologous proteins in T. gondii and P. falciparum but no clear homologous proteins in H. sapiens, S. cerevisiae, or A. thaliana. Interestingly, [Fe-S] cluster assembly key proteins (ISCS, ISCU1, and SSQ1) were identified among the candidate proteins, suggesting that NcGRX5 may be involved in [Fe-S] protein synthesis. In addition, the functions of other identified candidate proteins were found to be involved in TCA cycle, electron transport chain (ETC) complex, solute transport, redox, translation, and unknown functions.

### Interaction of NcGRX5 with ISCS and ISCU1 proteins.

**FIG 8:**
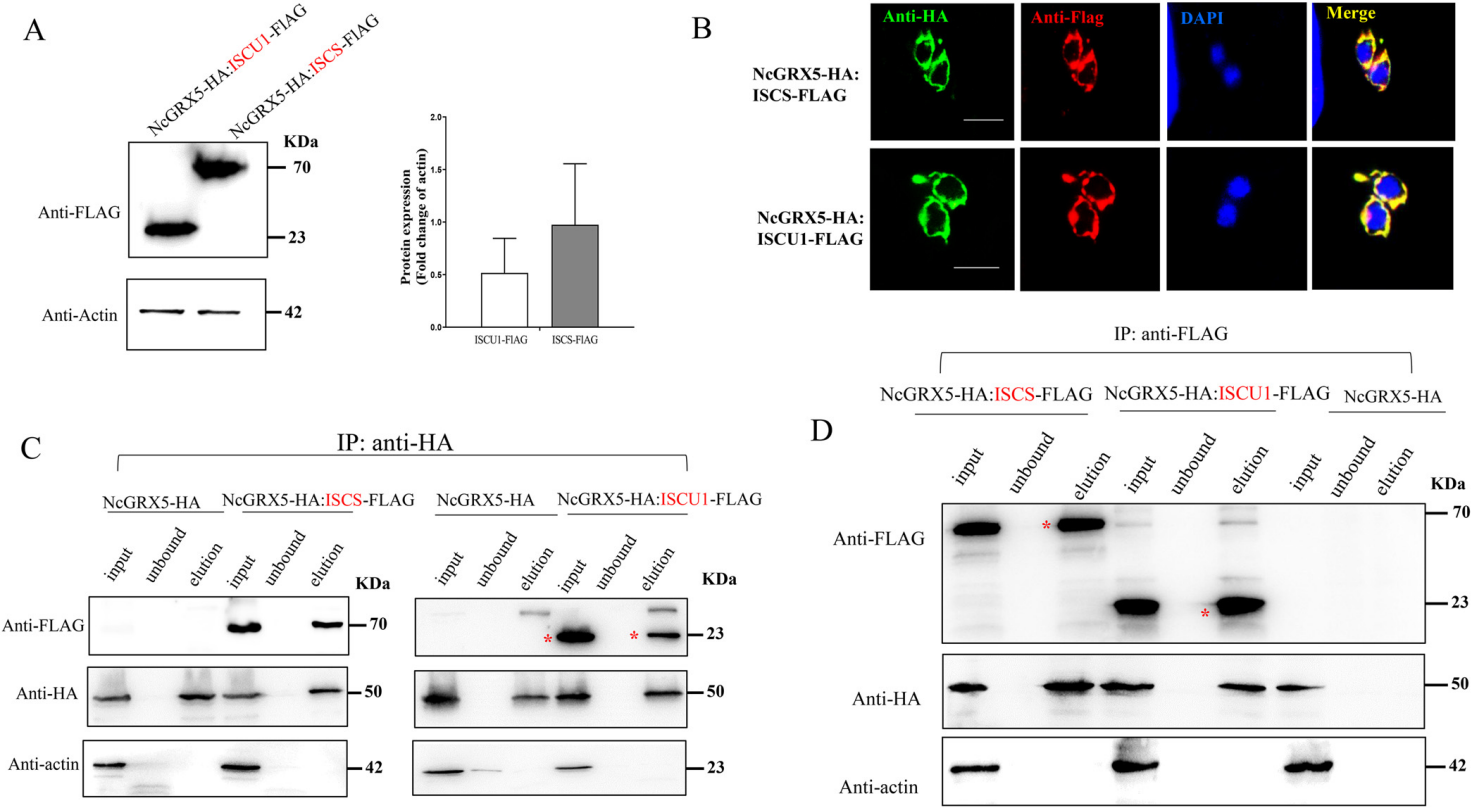
Interaction of NcGRX5 with [Fe-S] cluster synthesis proteins ISCS and ISCU1. (A) Tagging of the 3′ terminus of ISCS and ISCU1 with a FLAG tag in NcGRX5-HA parasites. Western blotting was performed to confirm successful addition of FLAG tags to the corresponding endogenous proteins. Actin was used as a control. The expression levels of FLAG-tagged proteins (ISCS and ISCU1) were quantitatively evaluated by ImageJ based on two independent experiments. Error bars represent the standard error (*n *= 2). (B) IFA images showing FLAG-tagged proteins (red) colocalized with NcGRX5 (green). Bar, 5 μm. (C, D) Performance of immunoprecipitation (IP) of proteins from NcGRX5-HA, NcGRX5-HA:ISCS-FLAG-, and NcGRX5-HA:ISCU1-FLAG-expressing strains using HA magnetic beads (C) and (D) FLAG magnetic beads. The input, unbound, and eluate fractions of the immunoprecipitation assay were detected using HA, FLAG, and actin antibodies. Two independent experiments were performed. Red asterisks represent the target proteins.

In eukaryotes, the [2Fe-2S] cluster is assembled by a complex consisting of the cysteine desulfurase Nfs1-Isd11-ACP1 and the ISCU scaffold. The assembled [2Fe-2S] cluster is rapidly transferred from the ISCU scaffold to a downstream transfer protein (GRX5) via a chaperone/cochaperone system. The [2Fe-2S] cluster was then inserted into [2Fe-2S] target proteins by GRX5 (8, 25, 26). To verify the involvement of NcGRX5 in [Fe-S] protein assembly and transport, we selected three proteins associated with iron-sulfur cluster assembly and transport (cysteine desulfurase ISCS [homologous to H. sapiens Nfs1], scaffold ISCU1 protein [homologous to H. sapiens ISCU scaffold], and HSP70 SSQ1 [homologous to H. sapiens HSP70 and S. cerevisiae HSP70 SSQ1]) from among the candidate interacting proteins used for coimmunoprecipitation (co-IP) verification (Fig. 7E). Parasites expressing ISCS-FLAG:NcGRX5-HA, ISCU1-FLAG:NcGRX5-HA, and SSQ1-FLAG:NcGRX5-HA were obtained by transferring the ISCS-FLAG, ISCU1-FLAG, and SSQ1-FLAG plasmids into the NcGRX5-HA parasite. Immunoblotting confirmed the detection of the corresponding tags in each strain (Fig. 8A; Fig. S3A). IFA detection confirmed that all three candidate proteins colocalized with NcGRX5 (Fig. 8B; Fig. S3B). Previous studies have shown that Plasmodium carries two scaffolding proteins (ISCU1 and ISCU2) (27). By comparing homologues, we found that N. caninum also harbors the ISCU2 gene. Hence, the C-terminal FLAG epitope was introduced into ISCU2 to confirm its mitochondrial localization (Fig. S3A and B).

NcGRX5-HA was then immunoprecipitated from ISCS-FLAG:NcGRX5-HA, ISCU1-FLAG:NcGRX5-HA, ISCU2-FLAG:NcGRX5-HA, and SSQ1-FLAG:NcGRX5-HA strains using antibodies against HA. The input, unbound, and eluted fractions were examined by immunoblotting using antibodies against FLAG, HA and actin, respectively. NcGRX5-HA parasites were used as controls. The co-IP results showed that ISCS and ISCU1, but not ISCU2 or SSQ1, precipitated with NcGRX5 (Fig. 8C; Fig. S3C). No significant band was detected in eluate fractions of the control parasites. Then, we immunoprecipitated ISCS and ISCU1 from the corresponding strains using an antibody against FLAG. The results showed that NcGRX5 coimmunoprecipitated with ISCS and ISCU1 (Fig. 8D). These results suggest that NcGRX5 interacts with ISCS and ISCU1 and is involved in [Fe-S] protein biosynthesis.

Previous studies in yeast have shown that the assembled iron-sulfur clusters are transferred from ISCU1 to GRX5 mediated via the mitochondrial HSP70 chaperone SSQ1 (10). The HSP70 SSQ1 candidate protein does not interact with NcGRX5 (Fig. S3C), but we found another protein (HSP70b) of N. caninum that is homologous to the human chaperone HSP70 in the ToxoDB database. Unexpectedly, our follow-up experiment revealed that HSP70b is located in the cytoplasm and does not interact with NcGRX5 (Fig. S3D). As a result, we were unable to identify related chaperone proteins involved in the mitochondrial [Fe-S] protein biosynthesis system of N. caninum.

## DISCUSSION

In eukaryotes, mitochondrial [Fe-S] proteins are involved in a wide range of critical biological processes, such as respiration (complexes I to III), the TCA cycle, β-oxidation of lipids, and lipoic acid synthesis (28). Mitochondrial [Fe-S] proteins are synthesized *de novo* on a complex platform comprising the scaffolding protein ISCU and the cysteine desulfurase NFS1-ISD11-ACP1 complex. Subsequently, the [2Fe-2S] cluster is specifically trafficked via the chaperone/cochaperone system to the monothiol GRX5, which then inserts the [2Fe-2S] target proteins via GRX5 (8, 25, 26). GRX5 can physically interact with the HSP70 chaperone SSQ1 at a site different from where it interacts with ISCU1 and receives [2Fe-2S] clusters from ISCU1 (8, 10). The crystal structure of the human homologue GLRX5 (GRX5) has [2Fe-2S] clusters that serve as bridges to aid GRX5 in transferring [Fe-S] proteins (9). The absence of the chaperone system membrane or GRX5 led to the accumulation of [Fe-S] clusters on ISCU1, which could have an impact on the downstream target [Fe-S] proteins, leading to the formation of [2Fe-2S] clusters (8). Although the deletion of GRX5 in S. cerevisiae was not lethal, it resulted in the accumulation of [Fe-S] clusters on ISCU1 and further affected downstream iron-sulfur proteins, suggesting that there may be an undiscovered compensation mechanism *in vivo.* In addition, this finding indicates that GRX5, which is similar to SSQ1 and Jac1, is also involved in the [Fe-S] cluster transferred by ISCU1 (28, 29). The deletion of GRX5 was previously shown to reduce the activities of [Fe-S] proteins and impair heme biosynthesis in yeast, zebrafish, and humans (30–33). Similarly, our results indicate that NcGRX5 is indeed not a lethal factor in N. caninum, but NcGRX5 deficiency significantly slows parasite growth *in vivo* and *in vitro*. These results suggest that NcGRX5 deficiency may not completely disrupt the transfer of [Fe-S] clusters but may affect the accuracy and efficiency of [2Fe-2S] cluster transport to downstream target proteins (28).

Mitochondria in apicomplexan parasites exist in the shape of a single lasso surrounding the nucleus of intracellular tachyzoites (17). Mitochondria are considered essential for parasite survival at all stages due to their central functions in various biological processes, such as energy generation and pyrimidine and heme biosynthesis (34–37). In this study, loss of NcGRX5 triggered the acquisition of aberrant morphology and a reduced number of mitochondrial cristae in N. caninum, with changes in cristae shape similar to those caused by ATP synthase dysfunction in yeast and T. gondii (18, 19, 38, 39). The inner membrane of mitochondria is divided into an inner boundary membrane (IBM), which runs parallel to the outer membrane and cristae formed by the inner membrane deep in the matrix. The ETC and ATP synthase complexes are distributed along cristae membranes with an overlapping profile and produce ATP for cell metabolism through oxidative phosphorylation (OXPHOS) (18, 40). The most important cristae morphology-regulating proteins are the mitochondrial-shaping proteins (MICOS complex, Drp1, PHB, and OPA1) and ATP synthase. Changes in cristae shape affect respiratory chain complex assembly and stability, as well as mitochondrial ETC efficiency, and further impair cellular metabolism and growth (41). Similar to these previous studies, NcGRX5 deficiency reduced the expression of several mitochondrial ETC and TCA proteins, including ATP synthase subunits. In addition, NcGRX5 deficiency also changed the corresponding metabolic fluxes. These effects may be one of the reasons for the morphological changes in the mitochondrial cristae.

The expression levels of several ATP synthase subunits or relevant proteins (such as the ATP synthase F_0_ OSCP subunit, ATP synthase F_1_ δ subunit, ATP synthase-associated protein ASAP12, ICAP11, and ICAP12) were significantly downregulated in ΔNcGRX5 parasites compared to Nc1 parasites. Correspondingly, energy metabolite detection also significantly reduced ATP levels in ΔNcGRX5 parasites. Thus, the comparative proteomics and energy metabolite results suggest that deletion of NcGRX5 affects mitochondrial respiration not only by inhibiting protein expression but also by influencing the activity of mitochondrial respiratory proteins.

**FIG 9:**
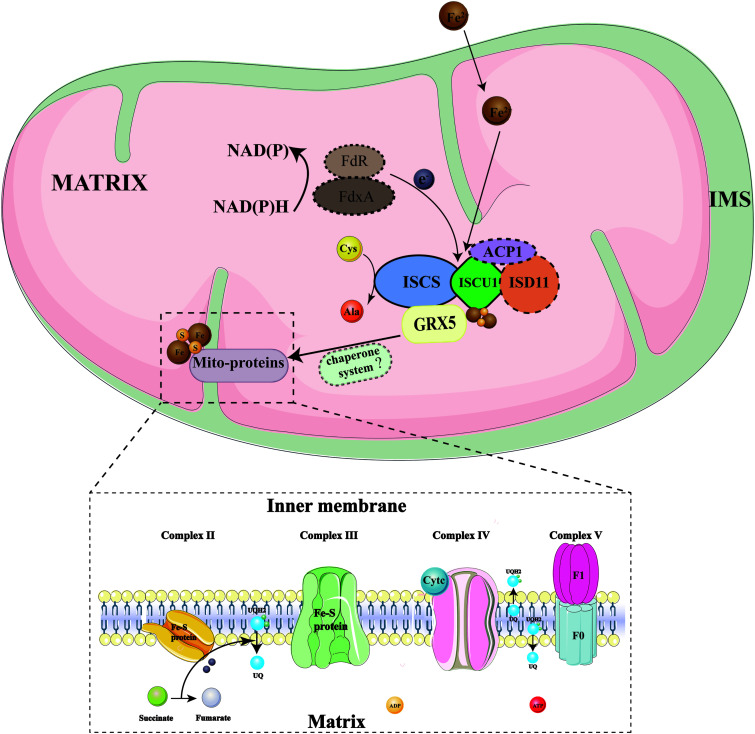
Diagram of the mitochondria iron-sulfur cluster (ISC) assembly pathway of N. caninum. The model is basically based on the ISC assembly pathway in yeast and human cells (8, 25, 26, 28). The ISC assembly pathway can be divided into three major steps. Initially, the electron (e^−^) is transferred from NADPH *via* ferredoxin reductase (FdxR) to the ferredoxin FdX2, and the [2Fe-2S] cluster is assembled by a complex composed of the cysteine desulfurase ISCS-ISD11-ACP1 and the ISCU1 scaffold. The assembled [2Fe-2S] cluster is rapidly transferred from the ISCU1 scaffold to a downstream transfer protein (NcGRX5) *via* a chaperone/cochaperone system (unknown). The [2Fe-2S] cluster is then inserted into [2Fe-2S] target proteins via NcGRX5. The proteins in the dashed box represent proteins that have not been identified in N. caninum. The solid outlined boxes represent proteins that were identified and found to interact with NcGRX5 in this study.

Using the BioID method in this study, we identified 23 possible mitochondrial proteins that interact with NcGRX5 (20). In addition, two identified iron-sulfur cluster biosynthesis proteins (ISCS and ISCU1) were shown to interact with NcGRX5. ISCS and ISCU1 were homologous to H. sapiens Nfs1 and H. sapiens ISCU scaffolding proteins, respectively, suggesting that NcGRX5 may be consistent with GRX5 in other eukaryotes by assembling complexes with [Fe-S] clusters ISCS-ISD11-ISCU1 and then transporting [Fe-S] clusters and then transporting iron-sulfur clusters (Fig. 9) (8, 25, 26, 28). We identified two possible proteins (HSP70 SSQ1 and HSP70b) in the ToxoDB database that may be homologous to human chaperone protein HSP70 (yeast HSP70 ssq1). HSP70 SSQ1, located in mitochondria, was identified among 23 possible candidate interaction proteins. However, the results of the co-IP assay confirmed that HSP70 SSQ1 does not interact with NcGRX5. HSP70b is located in the cytoplasm and does not interact with NcGRX5 or promote [Fe-S] cluster transport. Therefore, we have not identified chaperone-related proteins involved in the mitochondrial [Fe-S] protein biosynthesis system in N. caninum. It is possible that the chaperone/cochaperone system proteins in [Fe-S] protein biosynthesis of N. caninum are not conserved among eukaryotes. Alternatively, the interaction between HSP70 SSQ1 and NcGRX5 *in vivo* may be transient or unstable, which makes co-IP difficult to detect.

The branched-chain α-ketoacid dehydrogenase (BCKDH) complex in T. gondii and Plasmodium berghei has been shown to functionally replace host cell mitochondrial pyruvate dehydrogenase (PDH), which is critical for intracellular growth and virulence (42). The BCKDH complex catalyzes the conversion of pyruvate to acetyl-CoA and further to glucose in the TCA cycle (42). In this study, we verified that NcGRX5 is associated with the BCKDH complex: (i) The proteomic results showed that the absence of NcGRX5 resulted in decreased expression of the BCKDH E2 subunit in N. caninum. (ii) Various metabolic fluxes in the TCA cycle of NcGRX5-deficient parasites were reduced, especially those of citrate and TPP (cofactors in the synthesis of acetyl-CoA from pyruvate). This reduction is similar to the result of a decrease in citrate and 2-hydroxyethyl-TPP (intermediate in the acetyl-CoA production from pyruvate), which is caused by the lack of BCKDH-E1a in T. gondii (42). (iii) The activity of [Fe-S] protein lipoyl synthase (LIAS) was reduced due to the inhibition of Nfs1 (ISCS in N. caninum) function, which reduced the levels of lipoate and further weakened the activity of PDH (28). NcGRX5 deficiency may reduce the efficiency of [Fe-S] cluster transport to downstream proteins, thus affecting the activity of the BCKDH complex.

## MATERIALS AND METHODS

### Ethical statement.

The animal experiments were performed in strict accordance with the recommendations of the Guide for the Care and Use of Laboratory Animals of the Ministry of Science and Technology of China. All experimental procedures were approved by the Institutional Animal Care and Use Committee of China Agricultural University (under the certificate of Beijing Laboratory Animal employee ID 18049). The mice were humanely euthanized by cervical dislocation after anesthetization.

### Parasites and cell culture.

The N. caninum wild-type (WT) strain (Nc1) and derived strains were grown *in vitro* by serial passage on in human foreskin fibroblasts (HFFs; ATCC, Manassas, VA, USA) using Dulbecco’s modified Eagle’s medium (DMEM) supplemented with 2% fetal bovine serum (FBS) at 37°C and 10% CO_2_.

### Bioinformatic analysis of NcGRX5.

The complete gene sequence of NcGRX5 (NCLIV_037620) was downloaded from ToxoDB (https://toxodb.org/toxo/). The ExPASy Proteomics Server (http://expasy.org/) and SMART (http://smart.embl-heidelberg.de/) were used to predict conserved domains and motif analysis. Amino acid sequence alignment was performed using Clustal X software version 1.83. Three-dimensional structural modeling was performed using the SWISS-MODEL server (http://swissmodel.expasy.org), and the model was based on the crystalline structure of H. sapiens GRX5 (PDB code 2WU). When superposed NcGRX5 to H. sapiens GRX5 by PyMOL, the root-mean-square deviation (RMSD) values were 0.075 Å.

### Generation of transgenic tachyzoite strains.

The EuPaGDT Library in the ToxoDB database was used to design the guide RNAs (gRNAs) in the corresponding gene-specific CRISPR-Cas9-edited plasmids. The plasmid construction of pCRISPR-CAS9-GRX5 was performed as previously described (43). Briefly, the Cas9 upstream and downstream fragments containing gRNA sequences were amplified and ligated with a seamless cloning kit (Vazyme Biotech, Co., Ltd., Nanjing, China).

The Nc1 strain was used for CRISPR-mediated gene deletion and epitope tagging, as described previously (13). To generate the NcGRX5-deficient strain (ΔNcGRX5), the corresponding gene loci were disrupted using CRISPR-Cas9, and dihydrofolate reductase (DHFR) was inserted as a resistance selection tag. The 3′- and 5′-flanking sequences of NcGRX5 from the genomic DNA of Nc1 parasites were amplified. The DHFR expression cassette and vector backbone from the pLIC-HA-DHFR-NcGRA17 plasmid were amplified (43). All four fragments were ligated to form the p5’GRX5-DHFR-3′GRX5 plasmid using a seamless cloning kit. The p5’GRX5-DHFR-3′GRX5 plasmid and the corresponding CRISPR-Cas9 plasmid were linearized by PCR, transfected into Nc1 parasites, and selected with pyrimethamine *in vitro*. The monoclonal screening was carried out by a limited dilution method *in vitro*. The monoclonal parasites were identified by PCR followed by sequencing.

To complement the NcGRX5-deficient parasites, the UPRT gene was disrupted by a UPRT-specific CRISPR-Cas9 plasmid and replaced with the tubulin promoter-GRX5-3×HA sequence as described previously (13). p5′UPRT-Tubulin promoter-DHFR-GRX5-HA-3′UPRT and CRISPR/Cas9-UPRT plasmids were cotransfected into ΔNcGRX5 parasites. Fluorodeoxyribose (FUDR) was used for resistance selection. To generate the NcGRX5 overexpression strain (NcGRX5 OE), p5′UPRT-Tubulin promoter-DHFR-GRX5-HA-3′UPRT and CRISPR/Cas9-UPRT plasmids were cotransfected into Nc1 cells and selected with pyrimethamine. The resulting plasmid was linearized by PCR and transferred into ΔNcGRX5 parasites with a UPRT-specific CRISPR-Cas9 plasmid and selected with FUDR *in vitro*. The monoclonal screening was carried out by a limited dilution method *in vitro*. The monoclonal parasites were identified by PCR followed by sequencing.

To obtain NcGRX5-HA parasites, we constructed a pLIC-HA-DHFR-NcGRX5 plasmid into which a 3× HA tag was inserted into the 3′ end of the NcGRX5 gene as previously described (13). To generate NcGRX5-BirA*-HA parasites, we amplified the coding sequence of BirA*-HA using the vector NcGRA17-BirA*-HA as a template. Subsequent construction methods were consistent with those used to create the NcGRX5-HA parasites. To locate the NcGRX5 interaction or adjacent proteins candidates identified by mass spectrometry (MS), a FLAG tag was added to each candidate gene by CRISPR-mediated 3′ flank C-terminal FLAG tagging (TOM40, ISCS, ISCU1, ISCU2, and SSQ1). The FLAG tag and chloramphenicol resistance gene (CmR) expression cassette were amplified and ligated to the 3′-flanking C terminus of target genes. The homologous recombination plasmid and the corresponding CRISPR-Cas9 plasmid were cotransfected into NcGRX5-BirA*-HA or ΔNcGRX5 parasites and screened with chloramphenicol *in vitro*. The monoclonal screening was carried out by a limited dilution method *in vitro*. The locations of the candidate proteins in the global proteome and redox proteome were determined using the same method described above.

### Immunoblotting and immunofluoresence assays.

For immunoblotting assays, parasites were collected and purified by filtration through a 5-μm filter membrane and lysed with radioimmunoprecipitation assay (RIPA) buffer (Huaxinbio, Beijing, China). The primary antibodies used were rabbit or mouse anti-FLAG (1:5,000, Sigma, USA), rabbit or mouse anti-HA (1:5,000, Sigma), mouse anti-actin (1:6,000), and anti-NcGRX5 (1:500). Secondary antibodies used were goat anti-mouse or rabbit (1:5,000; Invitrogen). Quantification of signals on the membranes was evaluated by ImageJ. Immunofluorescence assays (IFAs) were used for subcellular localization. Tachyzoites infected HFFs were fixed by 4% paraformaldehyde (PFA) followed by treatment in 0.25% Triton X-100. Primary antibodies used were rabbit or mouse anti-FLAG (1:500, Sigma), rabbit or mouse anti-HA (1:500, Sigma), and rabbit anti-SRS2 (1:400). Secondary fluorescein isothiocyanate (FITC)- or Cy3-conjugated antibodies were used for labeling. DNA was stained with Hoechst 33258 (Sigma, USA). The images were obtained using a Leica confocal microscope system (Leica, TCS SP52, Germany).

### Phenotypic assays.

**(i) Plaque assays.** Plaque assays were performed as previously described (44). The purified parasites were used to infect HFF monolayers seeded on 12-well plates (300 tachyzoites/well). After culturing undisturbed for 9 days, the infected HFFs were fixed with 4% PFA and stained with crystal violet. The cells in the plaque area were counted by pixel using Photoshop C6S software (Adobe, USA), and the data from three independent experiments were compiled.

**(ii) Invasion assay and intracellular replication assay.** An intracellular replication assay was performed to assess the number of parasites per vacuole 24 h after invasion, consistent with a previous description (13). Briefly, HFFs growing in 12-well plates seeded on coverslips were inoculated with 1 × 10^5^ parasites and continuously cultured for 24 h. Then, IFA using rabbit anti-SRS2 antibodies and Hoechst dye was performed to observe the proliferation of parasites. The parasites of each strain in vacuoles were quantified by counting at least 100 vacuoles using a fluorescence microscope (Olympus Co., Japan). For the invasion assay, the invasion percentage was calculated on the basis of the number of vacuoles per host cell. Three independent experiments were performed.

**(iii) Egress assay.** HFFs were infected with parasites for 36 h. Egress was triggered with treatment by 2 μM A23187 Ca^2+^ ionophore (Sigma, USA) for 3 min, and then, the parasites were fixed with PFA and observed with IFA. The ruptured vacuoles and unbroken vacuoles were counted on each slide to evaluate the egress rate. The ruptured vacuoles and unbroken vacuoles in 100 random visualization fields/slide were counted to evaluate the egress ratio. Three independent experiments were performed.

### N. caninum mouse infection.

BALB/c mice (five mice of each strain) were infected with 1 × 10^7^ parasites. Survival was evaluated for 60 days.

### Electron microscopy.

For ultrastructural analyses, infected HFF cells were fixed with 1% glutaraldehyde (Polysciences, Inc.) and 1% osmium tetroxide (Polysciences, Inc.) in 50 mM phosphate buffer at 4°C for 30 min. The samples were then processed as described previously (19). Ultrathin sections (70 nm thick) were prepared with a microtome (Leica EM UC6), double-stained with uranyl acetate and lead citrate, and examined with a transmission electron microscope (FEI Tecnai Spirit120kV).

### Biotinylation approaches (BioID).

**(i) Biotinylated protein capture.** HFFs were infected with Nc-GRX5-BirA* and Nc1 parasites and cultured in medium containing 160 μM biotin for 24 h at 37°C with 5% CO_2_. The biotinylated proteins were examined by IFA using streptavidin-FITC. The biotinylated proteins were captured as previously described (45). Briefly, HFFs infected with NcGRX5-BirA* or Nc1 parasites were grown in medium containing 160 μM biotin for 24 h. The parasites were then harvested and lysed in RIPA buffer (Huaxin Bio, Beijing, China) supplemented with complete protease inhibitor cocktail (Roche) for 30 min on ice. The biotinylated proteins were captured using streptavidin magnetic beads (Beaver, USA). Lysates were incubated overnight with 150 μL of magnetic beads at 4°C. After washing five times with RIPA buffer and three times with 8 M urea buffer (50 mM Tris-HCl [pH 7.4], 150 mM NaCl), the beads were separated using magnets, removed, and resuspended in RIPA buffer; 10 μL of these beads were used for SDS-PAGE and Western blot analysis with streptavidin-horseradish peroxidase (HRP). The remaining fraction of the beads was analyzed by mass spectrometry (Beijing Qinglian Biotech Co., Ltd., Shanghai, China).

**(ii) LC-MS/MS and label-free MS quantification.** Mass spectrometry analysis of the biotinylated proteins was performed as described previously (46). The samples were dissolved, reduced, alkylated, and digested by successive addition of dithiothreitol (DTT), iodoacetamide, and trypsin. The trypsin-digested samples were analyzed by LC-MS/MS, which was performed with a Q Exactive mass spectrometer (Thermo Scientific) that was coupled to an Easy nLC system (Thermo Fisher Scientific). Peptide searches were performed with Mascot software (Matrix Science) using the N. caninum protein database (ToxoDB-46_NcaninumLIV_AnnotatedProteins) concatenated to a database of common contaminants (UniProt Human database, trypsin, etc.). Mascot was used to perform a search with a fragment ion mass tolerance of 0.060 Da and a parent ion tolerance of 10.0 ppm.

### Possible interaction or adjacent candidate protein analysis.

The identified biotinylated proteins of N. caninum were annotated through ToxoDB searches. Possible mitochondrial proteins among these biotinylated proteins were screened by using T. gondii mitochondrial proteome data (20). Mitochondrial targeting signal predictions were performed using MitoProt and LOPIT (21, 22). The homology of the candidate proteins with known proteins in model organisms (T. gondii, P. falciparum, H. sapiens, S. cerevisiae, and A. thaliana) was aligned by HHpred using a hidden Markov model-based search (24). The candidate proteins were located by endogenously tagged FLAG tags using the CRISPR technique.

### Coimmunoprecipitation (co-IP).

Immunoprecipitation was performed as described previously (47). Parasites were collected and lysed in nondenaturing lysis buffer (Huaxin Bio, Beijing, China) and lysed by ultrasonication on ice. After centrifugation at 15,000 × *g* for 20 min, the supernatants were incubated with FLAG or HA magnetic beads for 2 h at 4°C. The beads were washed five times, and bound proteins were eluted with SDS-PAGE sample buffer. The samples were used for an interaction analysis by Western blotting using rabbit or mouse anti-FLAG, rabbit or mouse anti-HA, and mouse anti-actin as primary antibodies.

### Comparative proteomics.

Nc1 and ΔNcGRX5 parasites were cultured in HFFs and purified using a 5-μm filter membrane. The samples were minced individually with liquid nitrogen and lysed in lysis buffer containing 7 M urea, 2 M thiourea, and 0.1% CHAPS (3-[(3-cholamidopropyl)-dimethylammonio]-1-propanesulfonate), followed by 5 min of ultrasonication on ice. The lysate was centrifuged at 12,000 × *g* for 15 min at 4°C, and the supernatant was transferred to a clean tube. Protein concentration was determined by Bradford protein assay. Trypsin (Promega, USA) was reconstituted in 50 mM acetic acid. The digestion was terminated by acidification (3 μL of TFA was added and incubated at 37°C for 45 min) and centrifugation (15,000 × *g* for 15 min).

Shotgun proteomics analyses were performed using an EASY-nLC 1200 ultra-high-performance liquid chromatography (UHPLC) system (Thermo Fisher) coupled with an Orbitrap fusion mass spectrometer (Thermo Fisher) operating in data-dependent acquisition (DDA) mode. The analytical column was purchased from Thermo Fisher (75-μm inner diameter; 150-mm length; packed with C-18 resin). The Orbitrap Fusion mass spectrometer was operated in the data-dependent acquisition mode using Xcalibur3.0 software, and a single full-scan mass spectrum was obtained with the Orbitrap (350 to 1,550 *m*/*z*, 120,000 resolution) followed by 3-s data-dependent MS/MS scans in the ion routing multipole with the normalized collision energy (HCD) of 38%.

### Energy metabolism analysis.

Metabolomic analyses were performed at Shanghai Applied Protein Technology Co., Ltd. (Shanghai, China). Parasites were collected and immediately frozen in liquid nitrogen and stored at −80°C until shipment. The samples were thawed at 4°C and mixed with 1 mL of cold methanol:acetonitrile:H_2_O (2:2:1, vol/vol/vol). The homogenate was sonicated at low temperature and centrifuged for 20 min (14,000 × *g*, 4°C). The supernatant was dried in a vacuum centrifuge. For LC-MS analysis, the samples were redissolved in 100 μL of acetonitrile/water (1:1, vol/vol), adequately vortexed, and then centrifuged (14,000 rpm, 4°C, 15 min). The supernatants were collected for LC-MS/MS analysis using an UHPLC instrument (1290 Infinity LC, Agilent Technologies) coupled to a QTRAP (AB Sciex 5500). For HILIC separation, the samples were analyzed with an ACQUITY UPLC BEH Amide column (2.1 × 100 mm, 1.7 μm, Waters MS Technologies, Manchester, UK). The quality control (QC) samples, prepared from pooled samples, were loaded onto the column at regular intervals in the analysis sequence to monitor the precision and stability of the method during its operation. In MS/MS analysis (MRM), for QC samples, a relative standard deviation (RSD) < 30% indicates that the metabolite is relatively stable and less influenced by the matrix effect. The quantitative value of each metabolite can reflect a difference. The quantitative result obtained with an RSD > 30% metabolite is not recommended for use as a reference. Each analysis was performed four times. MultiQuant software was used to identify the chromatographic peak areas and retention times. The AA standard correct retention time was used to identify the metabolites.

### Statistical analysis.

Graphs were created, and statistical analyses were conducted using Graph Pad Prism (San Diego, CA). The graphs represent the means, and error bars represent standard errors of means. All data were analyzed with the two-tailed Student’s *t* test and two-way analysis of variance (ANOVA). The *P* values are represented by asterisks in the figures as follows: *, *P < *0.05; **, *P < *0.01; and ***, *P < *0.001. We consider all *P* values <0.05 to be significant.

### Data availability.

The mass spectrometry proteomics data have been deposited to the ProteomeXchange Consortium (http://proteomecentral.proteomexchange.org) via the iProX partner repository with the data set identifier  PXD037871.
