# Supplementary material for: Regulation of Mitochondrial Energy Metabolism by Glutaredoxin 5 in the Apicomplexan Parasite Neospora caninum
Source: Microbiol Spectr. 2022 Dec 21;11(1):e03091-22. doi: 10.1128/spectrum.03091-22 (PMC9927405; doi:10.1128/spectrum.03091-22)
Supplement: Supplemental file 4 — Supplemental material. Download spectrum.03091-22-s0004.pdf, PDF file, 1.9 MB [file spectrum.03091-22-s0004.pdf]

Supplementary Materials:

Supplementary Figure S1: (A) Strategy for constructing NcGRX5-HA parasites. (B) PCR identification of ΔNcGRX5 parasites.

Supplementary Figure S2. The comparative proteome of ΔNcGRX5 and Nc1. (A) Volcano plots showing log2 protein ratios vs $-\log_2$ p values for the global proteome in ΔNcGRX5 compared to Nc1 parasites. (B) Gene Ontology (GO) analysis of down-regulated proteins in ΔNcGRX5 compared to Nc1 parasites based on molecular function, (C) cellular components, and (D) biological processes.

Supplementary Figure S3. Non-interaction of NcGRX5 with two putative HSP70 SSQ1 proteins. (A) Tagging of the 3'-terminus of SSQ1 and ISCU2 with a FLAG-tag in NcGRX5-HA parasites. Western blotting was performed to confirm successful addition of FLAG tags to the corresponding endogenous proteins. Actin was used as a control. The expression levels of FLAG-tagged proteins (SSQ1 and ISCU2) were quantitatively evaluated by ImageJ based on two independent experiments. Error bars represent the standard error (n=2). (B) IFA displaying FLAG-tagged proteins (red) co-located with NcGRX5 (green). Scale bar is 5 μm. (C) Performance of immunoprecipitation of proteins from NcGRX5-HA, NcGRX5-HA:ISCU2-FLAG, and NcGRX5-HA: SSQ1-FLAG strains using HA magnetic beads. The input, unbound and eluate fractions of the immunoprecipitation assay were detected using HA, FLAG and actin antibodies. Two independent experiments were performed. Red asterisks represent the target proteins. (D) Visualization of the location of HSP70b (green) with anti-FLAG and SRS2 (red) as parasite shape markers.

Supplementary Dataset 1: Comparative proteomic dataset of ΔNcGRX5 and Nc1 parasites.

Supplementary Dataset 2: Energy metabolic fluxes of ΔNcGRX5 and Nc1 parasites.

Supplementary Dataset 3: LC–MS hits of NcGRX5 proximal proteins using BioID.

Fig S1

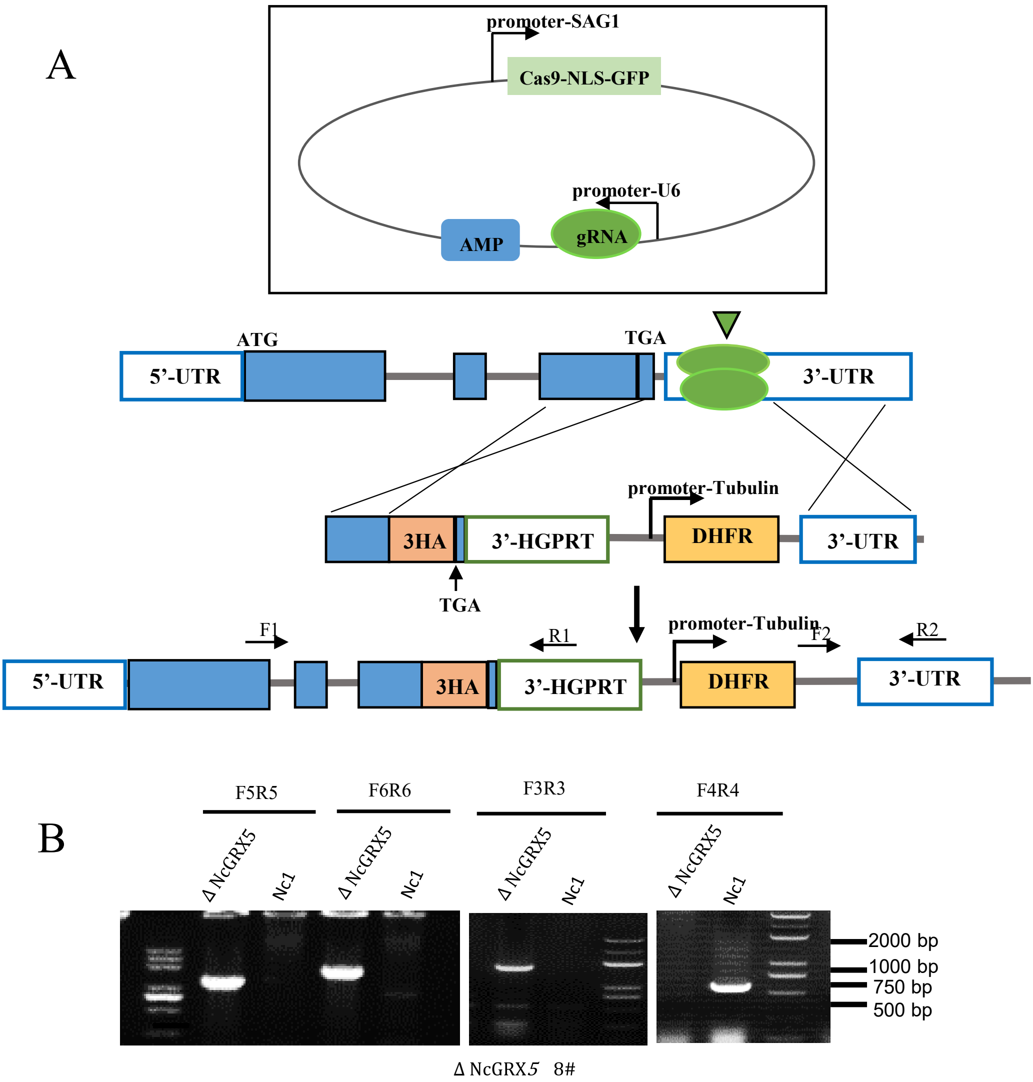
A
promoter-SAG1
Cas9-NLS-GFP
promoter-U6
AMP
gRNA
ATG
TGA
5'-UTR
3'-UTR
promoter-Tubulin
3HA
3'-HGPRT
DHFR
3'-UTR
TGA
F1
R1
promoter-Tubulin
F2
R2
5'-UTR
3HA
3'-HGPRT
DHFR
3'-UTR
B
F5R5
F6R6
F3R3
F4R4
Δ NcGRX5
Nc1
Δ NcGRX5
Nc1
Δ NcGRX5
Nc1
Δ NcGRX5
Nc1
2000 bp
1000 bp
750 bp
500 bp
Δ NcGRX5 8#

Fig S2

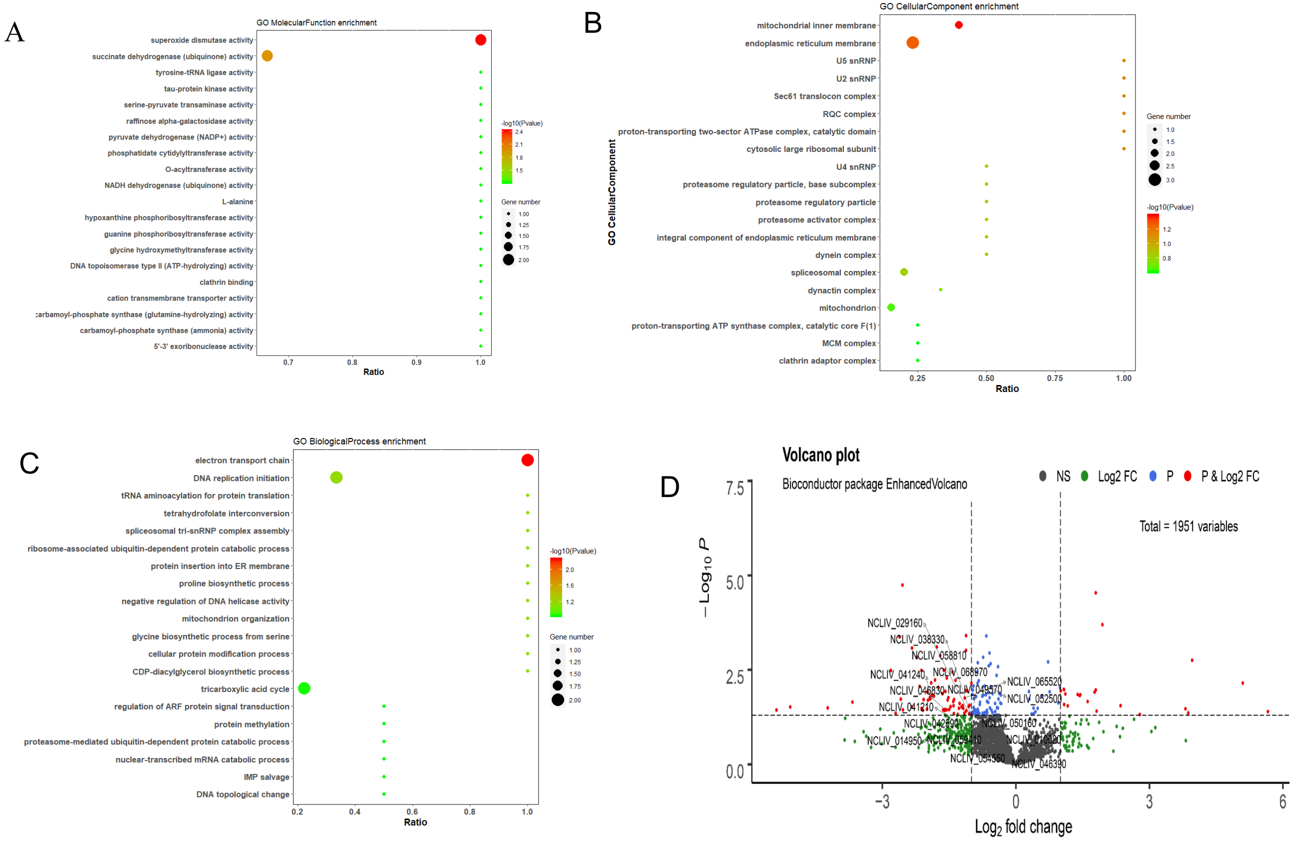

A
GO MolecularFunction enrichment
superoxide dismutase activity
succinate dehydrogenase (ubiquinone) activity
tyrosine-tRNA ligase activity
tau-protein kinase activity
serine-pyruvate transaminase activity
raffinose alpha-galactosidase activity
pyruvate dehydrogenase (NADP+) activity
phosphatidate cytidylyltransferase activity
O-acyltransferase activity
NADH dehydrogenase (ubiquinone) activity
L-alanine
hypoxanthine phosphoribosyltransferase activity
guanine phosphoribosyltransferase activity
glycine hydroxymethyltransferase activity
DNA topoisomerase type II (ATP-hydrolyzing) activity
clathrin binding
cation transmembrane transporter activity
carbamoyl-phosphate synthase (glutamine-hydrolyzing) activity
carbamoyl-phosphate synthase (ammonia) activity
5'-3' exoribonuclease activity
0.7
0.8
0.9
1.0
Ratio
-log10(Pvalue)
2.4
2.1
1.8
1.5
Gene number
1.00
1.25
1.50
1.75
2.00
B
GO CellularComponent enrichment
GO CellularComponent
mitochondrial inner membrane
endoplasmic reticulum membrane
U5 snRNP
U2 snRNP
Sec61 translocon complex
RQC complex
proton-transporting two-sector ATPase complex, catalytic domain
cytosolic large ribosomal subunit
U4 snRNP
proteasome regulatory particle, base subcomplex
proteasome regulatory particle
proteasome activator complex
integral component of endoplasmic reticulum membrane
dynein complex
spliceosomal complex
dynactin complex
mitochondrion
proton-transporting ATP synthase complex, catalytic core F(1)
MCM complex
clathrin adaptor complex
0.25
0.50
0.75
1.00
Ratio
Gene number
1.0
1.5
2.0
2.5
3.0
-log10(Pvalue)
1.2
1.0
0.8
C
GO BiologicalProcess enrichment
electron transport chain
DNA replication initiation
tRNA aminoacylation for protein translation
tetrahydrofolate interconversion
spliceosomal tri-snRNP complex assembly
ribosome-associated ubiquitin-dependent protein catabolic process
protein insertion into ER membrane
proline biosynthetic process
negative regulation of DNA helicase activity
mitochondrion organization
glycine biosynthetic process from serine
cellular protein modification process
CDP-diacylglycerol biosynthetic process
tricarboxylic acid cycle
regulation of ARF protein signal transduction
protein methylation
proteasome-mediated ubiquitin-dependent protein catabolic process
nuclear-transcribed mRNA catabolic process
IMP salvage
DNA topological change
0.2
0.4
0.6
0.8
1.0
Ratio
-log10(Pvalue)
2.0
1.6
1.2
Gene number
1.00
1.25
1.50
1.75
2.00
D
Volcano plot
Bioconductor package EnhancedVolcano
NS
Log2 FC
P
P & Log2 FC
Total = 1951 variables
$-Log_{10}$ P
7.5
5.0
2.5
0.0
NCLIV_029160
NCLIV_038330
NCLIV_058810
NCLIV_041240
NCLIV_068970
NCLIV_046830
NCLIV_049570
NCLIV_065520
NCLIV_052500
NCLIV_041210
NCLIV_042590
NCLIV_050160
NCLIV_014950
NCLIV_059410
NCLIV_010020
NCLIV_054550
NCLIV_046390
-3
0
3
6
Log_2 fold change

Fig S3

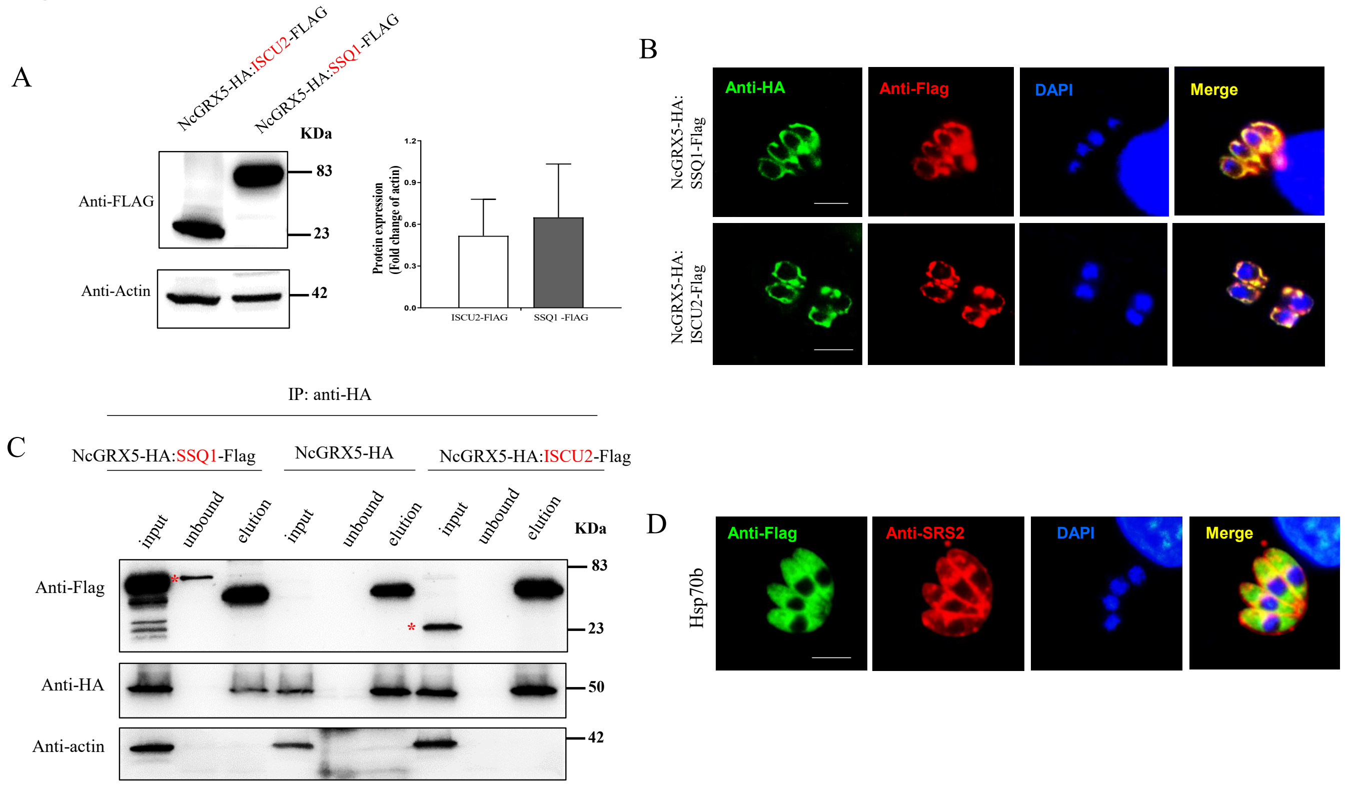
A
NcGRX5-HA:ISCU2-FLAG
NcGRX5-HA:SSQ1-FLAG
KDa
Anti-FLAG
Anti-Actin
83
23
42
Protein expression
(Fold change of actin)
1.2
0.9
0.6
0.3
0.0
ISCU2-FlAG
SSQ1 -FlAG
B
Anti-HA
Anti-Flag
DAPI
Merge
NcGRX5-HA:
SSQ1-Flag
NcGRX5-HA:
ISCU2-Flag
IP: anti-HA
C
NcGRX5-HA:SSQ1-Flag
NcGRX5-HA
NcGRX5-HA:ISCU2-Flag
input
unbound
elution
input
unbound
elution
input
unbound
elution
KDa
Anti-Flag
Anti-HA
Anti-actin
83
23
50
42
D
Anti-Flag
Anti-SRS2
DAPI
Merge
Hsp70b
